# Promising Role of Silk-Based Biomaterials for Ocular-Based Drug Delivery and Tissue Engineering

**DOI:** 10.3390/polym14245475

**Published:** 2022-12-14

**Authors:** Shahid Ud Din Wani, Mubashir Hussain Masoodi, Surya Prakash Gautam, H. G. Shivakumar, Sultan Alshehri, Mohammed M. Ghoneim, Prawez Alam, Faiyaz Shakeel

**Affiliations:** 1 Department of Pharmaceutical Sciences, Faculty of Applied Science and Technology, University of Kashmir, Srinagar 190006, India; 2 Government College of Pharmacy, Kangra 176047, India; 3 Department of Pharmaceutics, College of Pharmacy, JSS Academy of Technical Education, Noida 201301, India; 4 Department of Pharmaceutical Sciences, College of Pharmacy, AlMaarefa University, Ad Diriyah 13713, Saudi Arabia; 5 Department of Pharmacy Practice, College of Pharmacy, AlMaarefa University, Ad Diriyah 13713, Saudi Arabia; 6 Department of Pharmacognosy, College of Pharmacy, Prince Sattam Bin Abdulaziz University, Al-Kharj 11942, Saudi Arabia; 7 Department of Pharmaceutics, College of Pharmacy, King Saud University, Riyadh 11451, Saudi Arabia

**Keywords:** *Bombyx mori*, ocular drug delivery, silk fibroin, macromolecules, tissue engineering

## Abstract

Silk is a wonderful biopolymer that has a long history of medical applications. Surgical cords and medically authorised human analogues made of silk have a long history of use in management. We describe the use of silk in the treatment of eye diseases in this review by looking at the usage of silk fibroin for eye-related drug delivery applications and medication transfer to the eyes. During this ancient art endeavour, a reduced engineering project that employed silk as a platform for medicine delivery or a cell-filled matrix helped reignite interest. With considerable attention, this study explores the present usage of silk in ocular-based drug delivery. This paper also examines emerging developments with the use of silk as a biopolymer for the treatment of eye ailments. As treatment options for glaucoma, diabetic retinopathy, retinitis pigmentosa, and other retinal diseases and degenerations are developed, the trans-scleral route of drug delivery holds great promise for the selective, sustained-release delivery of these novel therapeutic compounds. We should expect a swarm of silk-inspired materials to enter clinical testing and use on the surface as the secrets of silk are unveiled. This article finishes with a discussion on potential silk power, which adds to better ideas and enhanced ocular medicine delivery.

## 1. Introduction

Silk fabric has been transported for centuries due to its lightness and texture [1]. The capability of releasing silk fabric has been altered [1]. It has been claimed that the medicinal application of silk has altered [1,2], particularly in diverse operations such as eye surgery. Our ability to employ direct and indirect techniques to assemble silk with critical elements has resulted in a flood of possible applications [3,4]. Recent updates explain current and rising trends in biomedical silk (composite) and advanced manufacturing [1,2,3,4].

**Figure 1:**
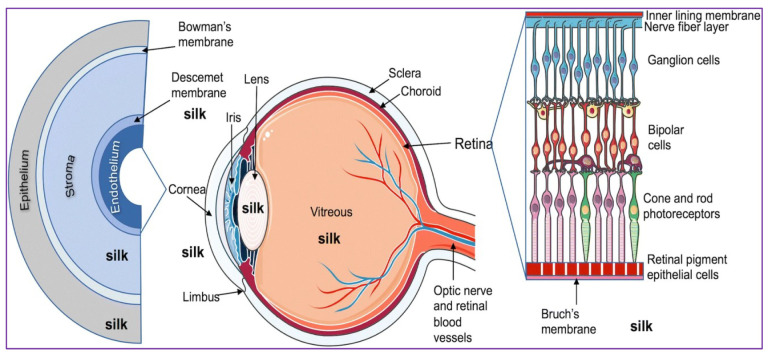
A schematic of a human eye displaying the expanded layers and layers of the retina. Silk fibroin figures obtained indicate the ocular regions where the silk was investigated. Image reproduced from reference [10], which was published under a creative common attribution (CC BY) license.

The eye is found and protected within the skull by tightening up muscles; thus, the spaces around the eye provide complete areas that can perform as loading points for the assembly of products such as silk hydrogels (as long as the view is not disturbed and their framework does not physically restrict the spinning of the ball). Building materials are frequently transferred beneath the orbital fascia, similar to the conjunctiva, and stored within the area between the world and therefore the skull (head injection) or within the ball. Subconjunctival injections are a common clinical practise among nursing personnel [5]; for example, therapy of progressive glaucoma is routinely assessed by subconjunctival injection. As a result, patient rates of interest may be substantially higher [6]. A good overview of the usage of this subtenon technique in anaesthesia is provided by Canvan and colleagues [7]. Typically, 2.0 mL quantities are supplied with ease. It may be required to keep the depots too far apart to avoid wiping techniques, although this lowers infiltration and disrupts the system. A retrobulbar injection, similar to that used after a football injection, has also been developed over time [8,9]. Cosmetic surgeons’ attention to the three-dimensional face was enhanced by accidental awareness, with claims that autologous oil injections might induce irreparable blindness due to occlusion. In an emergency, several injectable biomaterials, such as hyaluronate, are frequently shortened. Taking such efforts to properly halt the matrix in an emergency is a rare and important characteristic. Finally, the amount of space available is surprising: a full peribulbar injection with internal lubricants is capable of holding substantial volumes—often 6 to 12 mL. We describe the use of silk in the treatment of eye diseases in this review by looking at the usage of silk fibroin for eye-related drug delivery applications and medication transfer to the eyes (Figure 1) (Table 1) [1,10].

## 2. Silk Structure

The cocoons of *Bombyx mori* worms are made up of two proteins: silk fibroin and sericin. Heavy chains, light chains, and glycoprotein P25 make up the silk fibroin protein. The heavy chain of silk fibroin has a molecular weight of roughly 350 kDa [16,17] whereas sericin is a single hydrophilic protein with a molecular weight of 20 to 400 kDa [18], and it is the most important unit in determining the strength of a silk fibre [19]. These gleaming circuits, which are embedded in the amorphous matrix, are made up of well-organised sheets. The highest frequency of the GAGAGS motif [19,20] might be a sufficient silicon sequence to contribute to those b sheets. Silk fibroin is made up of a variety of secondary structures, such as sheets, helices, and curves. As a result, mechanical stresses (such as shear and strain), chemical solutions (such as solvents, pH, and ions), and temperature may all affect its compliance [21,22]. There are three varieties of silk fibroin: *silk-I*, *silk-II*, and *silk-III*. The majority of *silk-II* is made up of sheets inside the second structure, whereas *silk-I* is largely made up of an α-helix. Traditional *silk-I* has a rather high liquid solubility, around 26–30 wt/% [23,24], followed by refined silk, which mixes in at this high concentration.

## 3. Biocompatibility and Biodegradation of Regenerated Silk Fibroin

Silk’s strength makes it an ideal polymer for biological applications like stitching and tissue engineering. The clinically recognised silk in Europe and North America are silk clothing [25], silk garments for treating skin diseases, and silk invasive tools (SERI^®^ Surgical Scaffold) [26]. These items employ in vivo spun *B. mori* fibre. Though, success has been reported in the usage of preassembled silicon utilising B-silk in a number of investigations [27]. The cocoons of silk are converted into fibroin stock by extraction, which has the same traditional qualities as silk threads [28]. To liberate sericin, silk cocoons are first mined in smaller pieces and cooked with an alkaline solution, such as sodium carbonate. High-grade silk fibres are soluble in chaotropic salts such as lithium bromide, urea, guanidine hydrochloride, or salt, and the action of silk fibroin is suspended. The salt in the silk fibroin solution is dissolved in water, resulting in a liquid silk solution. Differences in processing factors, such as degumming extraction time, might result in a composite of recycled silk fibroin compounds with different cellular weights. This also helps silk fibroin’s activity, which combines mechanical strength [29,30,31].

For efficacy or in other biopolymer components, the biocompatibility and biodegradation of renewable silk fibroin have been studied in vitro and in vivo [1,32,33]. A great deal of studies on rats and big animals should be taken into account. The biocompatibility of silk fibroin is varied in different forms, such as meshes [26], thin films [34,35], hydrogels [11,36,37], nanoparticles [38,39,40,41,42,43,44], and liquid solutions [45]. Because the material composition, processing circumstances, and synthetic backdrop are predicted to contribute to degrading their profile, silk fibroin does not generate substantial contradicting reactions, corroborating assertions that silk fibroin is an SF-related biopolymer.

## 4. Ocular Wound Healing

Around 50 million individuals worldwide suffer from corneal blindness each year, with an estimated 2.5 million eye injuries per year in the United States, resulting in severe environmental harm today [46]. Furthermore, it is estimated that 337 million individuals globally suffer from the depressed symptoms of dry eye each year, making it a substantial risk factor for ocular injury [47]. Eye drop compositions, such as steroids to decrease inflammation, antibiotics to prevent infection, and synthetic tears including ointments to improve comfort are used at this level of treatment for ocular damage and dry disease [10,48,49]. Although eye drops are convenient to apply and have a wide range of applications, they do not directly encourage tissue regeneration, may hinder wound healing with long-term usage, and only give a brief alleviation of symptoms [50]. Corneal homeostasis entails a slew of tightly controlled cell behaviours as well as a scientific sequence of systematic activities that necessitate corneal tissue care and enable diagnostics [51]. The corneal epithelium is the most sensitive tissue engaged in the selection of painful scars and illnesses from chemical, mechanical, and environmental stimuli produced by damage or surgery since the outer layer covers the ocular region [52]. Corneal diseases are excruciatingly painful, and severe ocular injuries can compromise the corneal tissue’s inherent ability to restore a healthy epithelial environment [53]. Chronic corneal disease and loss of vision from visual ocular acuity can occur as a result of this disorder [54]. Due to its high solubility of soluble materials and stability profile, it produces a combination of soluble fibres termed Silk-Derived Protein (SDP), which turns it into a state of viscosity [55].

**Figure 2:**
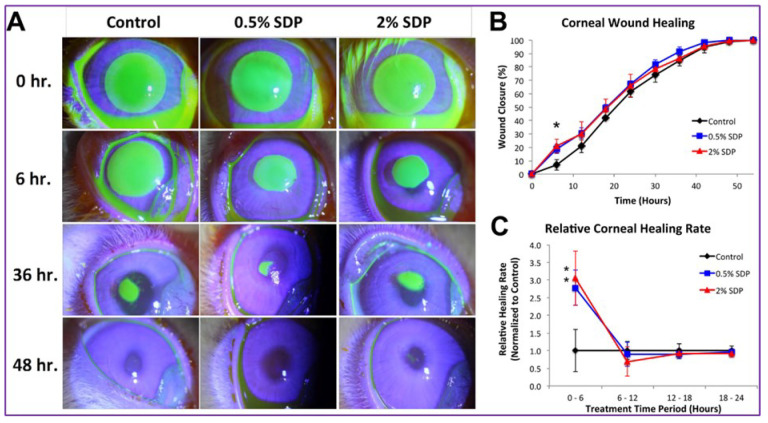
Treatment of rabbit ulcers with silk protein. Fluorescein signal (i.e., the site of the wound) within the presence of the areas mentioned in rabbits after epithelial rupture. (**A**) Representative post-surgical fluorescein staining, (**B**) summary of wound closure (percent over time), and (**C**) normalized healing rates between 6 and 24 h post debridement increasing in healing rate with both SDP concentrations. Image reproduced from reference [12], which was published under a CC BY license.

The attributes of silk fibroin regeneration in ocular tissue engineering are often used in a variety of ocular management systems, such as wound repair in the area of attention. In the wound area, the movement of epithelial cells is often followed by the replication and division of limbal stem cells to substitute injured tissue in ocular wound healing. Silk fibroin, in comparison to its role in tissue engineering, does not act as a specialised cell and develops in the wound-healing process; relatively, it acts as a therapeutic effect that brings healthy epithelial cells closer to the injured region. To initiate the in vitro healing process, epithelial cell transplantation, enlargement, and adhesion to the wound site were demonstrated using epithelial cells and wound examination [12,44]. The ability of silk protein to repair wounds was tested in a rabbit wound model [12] (Figure 2). The rabbit corneas were stripped of their epithelial layer and treated with silk protein containing 0.5 percent and 2.0 percent concentrate in the eye; the PBS drops acted as a complete spectrum, and in the treatment groups, the rabbits showed >95 percent cardiac fixation within 48 h of treatment, though the rate of correction was significantly higher after a silk protein treatment.

Silk fibroin wounds are considered to have therapeutic effects on dry skin due to their healing ability. This condition is characterised by the severe deterioration of the tear film, leading to injury to corneal epithelial cells and damage to connective tissue cells in the lower extremities and, eventually, impairment to corneal epithelial cells and injury of conjunctival shell cells [45,56]. The authors also looked at the effects of silica fibroin on tear production, corneal dysfunction, corneal vegetative cell division, conjunctival cell congestion, and inflammatory factors in the lacrimal gland using a dry-eye mouse model. In 12-week-old mice, dry eye conditions were implicated by immersing them in 30–40 percent humidity for 10 days and injecting them with scopolamine hydrobromide. Two experimental groups obtained a silk fibroin solution of 1–5 mg/mL and one control group received PBS in this study. The liquid silk fibroin solution found in B-fibres was used. Moreover, the whole building was made alive in an eye-catching way.

## 5. Silk Fibroin-Based Ocular Drug Delivery

The fibre content of fibroin binds reliably to hydrogel in response to the effect of a fine polymer delivery of ocular macromolecules, for example antibodies and other therapeutic proteins [31,57]. One example is the inclusion of bevacizumab in silk hydrogel, an antibody that prevents the formation of vascular endothelial protein in the treatment of macular degeneration by dehydration. The hydrogel strength of silk within ocular drug treatment, which includes sterile processing power, biocompatibility compatibility, and general strength, was demonstrated in vitreous rabbits and Dutch belts via intravitreal injections [35,58]. Because silk fibroin is frequently collected in aqueous solutions as a hydrogel, sterile solid fibre fibroin and bevacizumab are normally combined before sonication-induced gelation. Although the secret to creating second-generation hydrogel silk appears unachievable, Lovett and colleagues [11] have highlighted the immense power of silk hydrogel as a method for the administration of vitreous medicines. The silk hydrogel, for starters, was absolutely undetectable to the vitreous, placing the observer at risk. Second, because the capacity for silk hydrogel biodegradation in the vitreous is unclear, the findings in the literature are suspect because silk hydrogel deterioration might be attributable to shrinkage rather than disintegration. Furthermore, bevacizumab integrated in silica hydrogel resulted in a 90-day continuous-release period, compared to 30 days for commercial bevacizumab solution.

Lyogels are hydrogel compositions that have been lyophilised. The high quantity of silica fibroin also aids the quick release of lyogel, which regulates solvent penetration and, as a result, disrupts silica–antibody hydrophobic mixing. Guziewicz and colleagues, for example, discovered that increasing the saturation of silk fibroin within the genus lyogel decreased the concentrated release of the murine IgG1 antibody, as well as its total recovery [59]. This hydrophobic connection might be linked to a pH impact and a continuous ionic interaction that, albeit mild, increases antibody release and acquisition. The silk matrix can also harm essential methionine scans with a closed antibody, according to this study, because the oxidation of these particles increases as the density of silicon increases. Oxidation increases from 6% to 28% with 3% silk gel and up to 34% with 7% lyogel [59]. Despite the fact that the oxidation discovered during the study had no effect on the model protein’s biological function, such chemical alterations impair other therapeutic approaches and applications [60,61,62]. A data spokesperson for liposomes made of silk fibroin may be the attention-grabbing factor needed to promote the drug delivery system.

**Figure 3:**
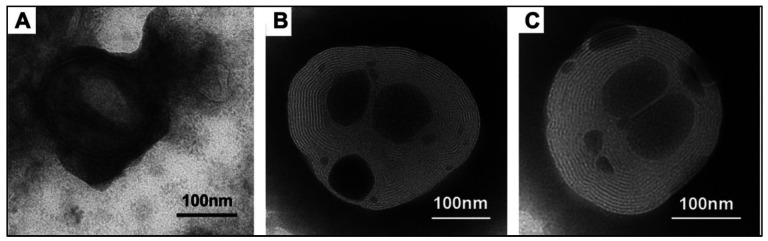
TEM images of drug-loaded liposomes (**A**) and SF-modified SL have a specific termination time: (**B**) 2 h; (**C**) 8 h. Image reproduced with permission from reference [13].

For the administration of topical ocular medicines, Dong et al. [13] created a liposomal structure connected with the unique excipient coating, silk fibroin (SF). Liposomes filled with liposomes were coated over regenerated silk fibroins (RSFs) with a specific melting point. In comparison to the regular liposome, SF-coated liposomes (SLs) were studied in terms of morphology, drug effectiveness, in vitro release, and in vitro corneal permeability. Human corneal epithelial cells were used for cell adhesion as well as cytotoxicity tests of SF and SL (HCEC). In addition, the researchers looked at whether SLs displayed continuous drug release and in vitro ibuprofen corneal penetration as compared to drug and liposome solutions. After 7 min of SF exposure, the cell fluoresced, and the pressure was steadily raised for 12 h without evident cytotoxicity. During a brief 15 min period, Nile power of the red fluorescence on SLs was detected, suggesting acceleration. Using SF-coated liposomes is also a viable strategy for the administration of ocular medicines due to their appealing shapes [63,64,65,66]. To measure particle size, liposomes loaded with ibuprofen and SL were filtered using TEM at 2 h SF and 8 h SF (Figure 3).

**Figure 4:**
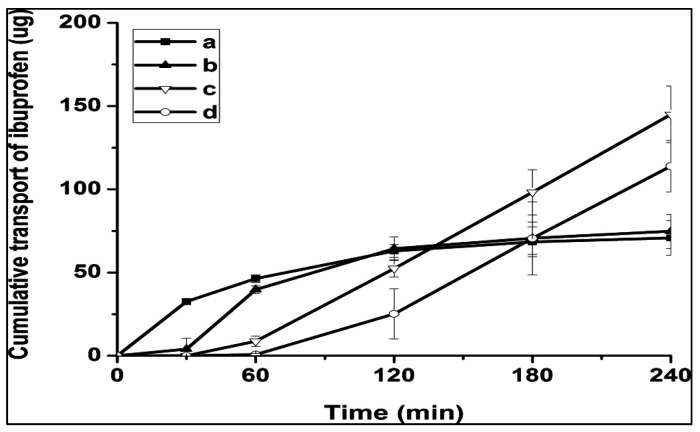
Ibuprofen (**a**), liposomes (**b**), and SL produced by 1.0 percent SF at various concentrations of in vitro corneal permeability: (**c**) 2 h; (**d**) 8 h. The data are shown as mean ± SD (*n* = 3). Image reproduced with permission from reference [13].

Most unilamellar liposomes with a touch of multilamellar liposomes with particles smaller than 200 nm were introduced using an ethanol injection approach. Many vesicular liposomes are collected by SF liposome coagulation to produce a vesicular liposome that displays the size of big particles in an ordered and filtered way. Additionally, Figure 4 shows the effects of an in vitro corneal permeation of liposomes and SL, and the delay time was fully detected by liposomal formation. The visible penetration coefficients (Papp × 10^7^) were 1.11 ± 0.12, 1.16 ± 0.23, 1.26 ± 0.19, and 1.23 ± 0.24 cm/s with a solution of ibuprofen liposomes in 2 h SLs and 8 h SLs.

**Figure 5:**
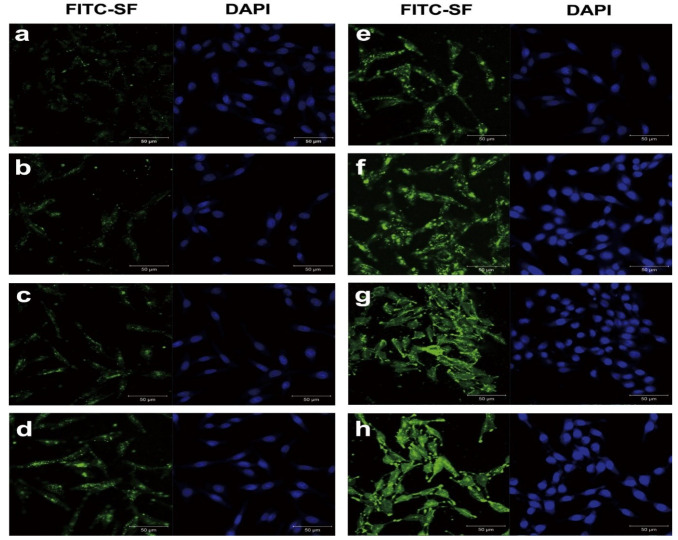
Direct HCEC images displayed in SF-labelled FITC: (**a**–**h**) stands for 7, 15, 30, 60, 120, 240, 480, and 720 min, respectively, at 37 °C. Scale bars represent 50 µm. Image reproduced with permission from reference [13].

Despite the fact that the Papp values were not significantly different, the in vitro corneal permeation curves for the drug solution, nonliposome, and SL gaps showed distinct behaviours. Drug transport from solution and unclosed liposomes takes approximately 2 h to reach bases, but drug loading from SLs increases rapidly after about 1 h, and SF coating improves and maintains penetration. SF-labelled FITC and Nile red liposomes were shown on HCEC and were obtained by confocal microscopy. The SF adherence behaviour in HCEC for a period of 12 h is shown in Figure 5. After 7 min of exposure to SF, an FITC green cell fluorescence appeared, and the fluorescence strength steadily rose until 12 h of incubation. The cellular absorption of SF nanoparticles by murine somatic cell carcinoma 4 cells and SF-coated liposomes by Keloid cells [67,68] was nearly equivalent to the quick and continuous attachment of SF to HCEC. SF-coated liposomes are a potential medication delivery technology for the eyes because of all of these characteristics.

Retinal and central vein closure, retinal vascular disease, diabetes, and glaucoma, all of which cause retinal and optic nerve damage, have recently become the most common causes of vision loss and blindness. ARMD affects around 50 million people worldwide, and the number of cases is predicted to rise in the next 20 years, indicating that it has become a major public health concern [69,70,71,72]. In general, medicine is administered as soon as possible via a number of methods [73,74]. Formal therapy is widely employed, but it has harmful side effects since it frequently fails to identify the eye due to its distinct physical and biological structure and inherent barrier to eye protection [75]. Several attempts have been undertaken to improve drug retention and sclerosis in order to improve the therapeutic impact [76,77,78,79,80,81,82,83,84]. A bioavailability discovery event and a controlled release system are frequently separated into two phases in these attempts. To improve bioavailability, researchers monitor viscosity enhancers [85], implant enhancers [86], active medicines [87], and portable technological devices such as iontophoresis [88] and ultrasounds [89]. Ultrasounds provide a number of advantages as a noninvasive therapy, including a rapid application procedure, dosage assessment, and interaction with other medication delivery systems. Several plants [90,91], chemicals, and delivery methods, such as liposomes [92], micro- or nanoemulsions [93], and tiny nanoparticles [94] are used in the controlled emission system. Nanoparticles are one of the most promising forms because they have the advantages of halving the drug’s half life, lowering high volume and toxicity, reducing volume frequency, boosting biocompatibility, and injecting a single drug molecule.

**Figure 6:**
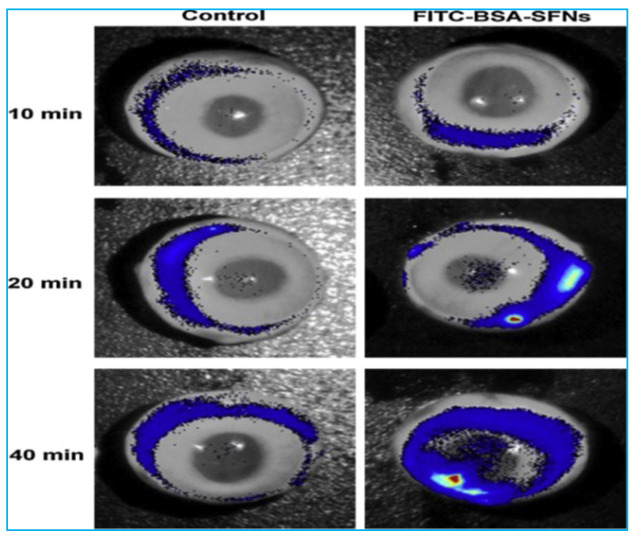
In vivo fluorescence of rabbit scleras attached to FITC-BSA-SFNs and a free FITC-BSA control solution for 10, 20, and 40 min. Image reproduced with permission from reference [14].

A transscleral ultrasound was used by Huang et al. [14] to deliver silk fibroin nanoparticles (SFNPs) for long-term medication delivery. The macromolecular protein model was fluorescein isothiocyanate bovine albumin (FITC-BSA, MW 66.45 kDa), and SFNPs were used as nanocarrier systems for complete drug delivery. In vitro transscleral investigations with ultrasound (1 MHz, 0.5 W/cm^2^, 5 min continuous wave) were performed on rabbit sclera that had been separated. The rabbit’s posterior eye region was evaluated for any adverse effects using a slit lamp and histology. Continuous extraction, bioadhesive qualities, and copermeation were used to create FITC-BSA-SFN. An ultrasound considerably enhanced the efficacy of the FITC-BSA-SFN entrance when compared to passive administration; however, it may cause harm to the ocular tissue or the particles themselves. External scleral tissues attach quickly and last a long time in the SFN delivery profile, followed by internal migration that lasts up to a week. Researchers are also continuing to look into in vivo mucoadhesion. Fluorescence imaging equipment was used to examine the in vivo distribution of fluorescence medication linked to rabbit sclera, as fluorescent light from FITC is generally generated at 525 nm. After the automated exclusion of the autofluorescence tissue from the visible sclera spectrum, the fluorescent light signal displayed a protein fluorescent sclera after FITC-BSA-SFN suspension, and the FITC-BSA control solution was shortened to the rabbit sclera region (Figure 6).

**Figure 7:**
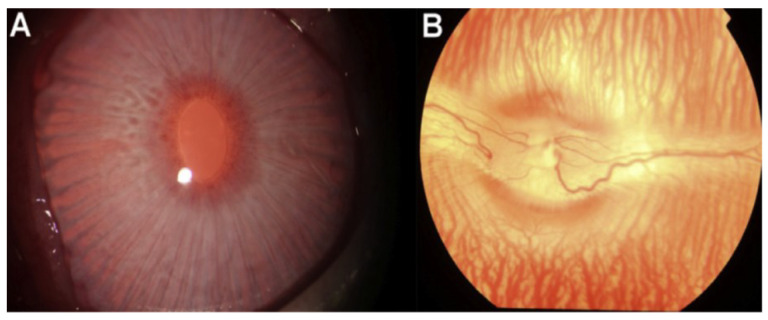
Visualisation of the light on the front part (**A**) as well as the rabbit eye (**B**) after the ultrasound scan. Image reproduced with permission from reference [14].

The fluorescent signal steadily grew, resulting in an increase in the adhesion area over time. At 10, 20, and 40 min after delivery, the FITC-BSA-SFNs group showed a stronger and more flexible adherence to the sclera than the control group, implying that SFNs aid medications in treating sclera effectively and efficiently. They also conducted more studies by taking X-ray pictures that revealed no difficulties inside the visual and anterior chambers, such as cerebral palsy, keratopathy, or synechia (Figure 7A). Funduscopic testing found no signs of vitreous opacity, endophthalmitis, retinal detachment, haemorrhage, or oedema in rabbit eyes after ultrasonography. The retina and optic nerve appeared to be healthy (Figure 7B). The temperature difference between before and after treatment of the scleral surface inside the ultrasonic application region was also 0.8 °C. Their initial study demonstrated that an ultrasound could be used to improve the transport of protein-rich nanoparticles to the sclera and to better understand subsequent release. SFNP formulations showed rapid adherence, effective breathing, and sustained medication release. SFNs are a promising carrier of ocular medication delivery because of all of these characteristics. To establish the feasibility of using a safe trans-scleral ultrasound to enhance the delivery of SFNPs loaded with protein medicines for clinical usage, further drug delivery research and in vivo investigations are needed.

## 6. Conclusions

Around 50 million individuals worldwide suffer from corneal blindness each year, with an estimated 2.5 million eye injuries per year in the United States, resulting in severe environmental harm today. For efficacy or in other biopolymer components, the biocompatibility and biodegradation of renewable silk fibroin have been studied in vitro and in vivo. Silk biopolymers have been used in tissue engineering for a few years, and their usage in drug research has just recently become popular. Silk fibroin is increasingly being used as an active biological polymer. We should expect a swarm of silk-inspired materials to enter clinical testing and use on the surface as the secrets of silk are unveiled. Despite the fact that silk has been used as a stitching material for thousands of years, the introduction of synthetic silk and the capacity to regenerate silk fibroin has opened up a world of silk functions in the treatment of eye disorders.

## 7. Possibilities for Improvement of Silk-Based Biomaterials for the Ocular Drug Delivery

As treatment options for glaucoma, diabetic retinopathy, retinitis pigmentosa, and other retinal diseases and degenerations are developed, the trans-scleral route of drug delivery holds great promise for the selective, sustained-release delivery of these novel therapeutic compounds. Many of the criteria for a competent biomaterial, such as a lack of direct cytotoxicity, are met by fibroin, and its long use in the human body has supported regulatory approval. However, significant questions remain unanswered. How much will choroidal blood flow restrict drug delivery across the sclera? How will transscleral pressure affect protein and macromolecule diffusion across tissues? Is it possible to develop a practical delivery system with pharmacokinetics compatible with long-term sustained-release delivery? Can regional scleral thickness differences be used to optimise trans-scleral delivery? How will drugs binding to the scleral extracellular matrix affect drug delivery and sustained release? Determining in vitro to in vivo correlations and scale-up needs are the next steps towards clinical applications.

## 8. Future Perspective

Although silk has been used for years as a stitching material, the introduction of synthetic silk, the advent of recombinant silks, and the ability to regenerate silk fibroin has opened up a world of silk functions in the treatment of eye disorders. Silk biopolymers have been studied for decades for tissue engineering applications, but its use in drug delivery applications has only recently emerged. Silk fibroin is becoming more popular as a biologically active polymer. As we continue to discover the secrets of silk, more silk-inspired materials will enter clinical testing and, eventually, clinical use.

## Figures and Tables

**Table 1:** Silk-based biomaterial delivery of therapeutics for ocular delivery.

S. No	Formulation	Outcome	Released Agents	Ref
01	Hydrogels	Sustained ocular delivery entails releasing the product over a period of 90 days. Biodegradation begins three months after intravitreal injection.	Bevacizumab	[11]
02	Silk-Derived Protein (SDP)	Improves the healing of rabbit corneal epithelial wounds.	Phosphate buffered saline (PBS)	[12]
03	Silk fibroin-coated liposomes (SLs)	Continuous and rapid adhesion and uptake of SF and SLs in cornea cells has been reported, with no detectable cytotoxicity during the experimental course.	Ibuprofen	[13]
04	Silk fibroin nanoparticles (SFNPs)	SFNP carriers for noninvasive transscleral administration of macromolecular protein drugs	Fluorescein isothiocyanate labelled bovine serum albumin	[14]
05	Silk fibroin nanoparticles	Bio-macromolecule delivery to the retina is improved.	Fluorescein isothiocyanate labelled bovine serum albumin (FITC-BSA)	[15]

## Data Availability

This study did not report any data.
